# Need of University Students to Study Man

**Published:** 1913-03

**Authors:** 


### Need of University Students to Study Man.

*Editor Texas Medical Journal, Austin, Texas.*

MY DEAR SIR: If I were able, I should be glad to write to every university student who is interested in the scientific and sociologic study of man, especially criminal, pauper and defective men. I trust, therefore, you will publish this letter, and I request each student to regard it as a personal letter to himself, whom I shall be pleased to help all I can, should he desire to devote his life to the fundamental study of social pathology.

I appeal to university students to direct their attention more to the scientific study of humanity. It is a cry to "Come over into Macedonia and help us." Let the university encourage students more to take up these subjects which have been so long neglected and in which there are great opportunities to aid humanity, directly, by scientific investigation of the causes of crime, pauperism and defectiveness, in order to prevent and lessen them through knowledge gained by first-hand study of the individuals themselves.

When a student chooses for his lifework a subject in the older branches of knowledge as physics, philosophy, philology, Greek, Latin, and natural history, he finds the field somewhat well developed; but not so in more recent sociological lines of research, as criminal anthropology (criminology, shorter term), and other cognate subjects, in which there is full opportunity for mental acumen and scientific ability of the highest character, to carry out most lofty purposes.

The question may arise as to what course of study will prepare one best for such work. I would suggest the following:

1. A two-years' course in psychology, especially laboratory work.

2. Medical studies to the extent of anatomy, physiology, general pathology, nervous diseases and insanity (especially clinical studies).

3. A practical course in craniology in the laboratory.

4. Facility in reading modern languages, especially German and French.

Thus social pathology, especially criminal anthropology, one of its branches, requires more extensive preliminary training than most subjects, for it involves the investigation of man both mentally and physically. Such training is synthetic, which in this age of specialism, is much needed. As such education is relatively new and experience in it as yet limited, it is difficult to designate a preparatory course. I have myself followed the course of study just indicated, but more extensively especially in medical lines, but such additional preparation might not be practicable for most students.

The enclosed leaflet, entitled "Study of Man," explains the work more fully, and I shall be glad to mail it to any student, gratis, who will send me his address. As I have said in this leaflet, "Criminals, paupers, mattoids, and other defectives, are social bacilli, which requires as thorough scientific investigation as the bacilli of physical diseases."

I beg leave to remain,

Most faithfully,

ARTHUR MACDONALD.

"The Congressional," Washington, D. C., January 21, 1913.

A LEADING EDITORIAL in the *American Journal of Surgery* (New York, February) says in substance:

"Like most investigators who have sought vaccines for bacterial infections, Friedmann came to the conviction that the most potent curative and immunizing powers lie in the living bacterial organism itself, and not in the dead organism, as used in the method of Wright and his school. After many years of observation and experiment, Friedmann finally obtained a stock of tubercle bacilli, which by repeated culture and passage through animals became entirely avirulent for the human organism. Under pressure, Friedmann admitted in the discussion that his bacillus was derived from one of the cold-blooded animals—the turtle.

"After demonstrating by injections into animals and even into himself that living vaccines derived from this bacillus were entirely harmless, he began to inject these vaccines into patients afflicted with various forms of tuberculosis. These injections are repeated at intervals of a few weeks, as many as half a dozen being sometimes administered.

"The remedy has now been used by Friedmann and his workers the past year or two in 1182 cases of pulmonary and surgical tuberculosis. Friedmann offers no statistics, he merely states that after one, two, or more injections, all cases of tuberculosis except those far advanced, are completely cured. As proof of his claims, he merely contented himself by presenting in the course of his address a number of cured cases, the recital of which is highly impressive.

"It is only a short step from cure to immunization, and Friedmann has already started on this problem on an extensive scale. He has thus far vaccinated 335 children, ranging from the newly-born to the age of 3 years. Most of these children had tuberculous surroundings, and although some of the children were injected over a year ago, in not one has tuberculosis developed.

"Friedmann's address is naturally tinged by copious optimism and enthusiasm and affords very little opportunity for a well-balanced judgment of his work. That is why we followed the discussion of his paper with perhaps greater interest than his original thesis. The expressions of those who took part in the discussion may be grouped in four classes; first, the enthusiastic; second, praising with reservations; third, mildly critical; fourth, expectantly conservative. It is interesting to note that, with one or two exceptions, those who praised the remedy were those who had actually used it. No one deliberately said that the remedy was of no avail. The largest part of the criticism came from the laboratory workers, such as Citron."

